# Characterization of the aldo-keto reductase 1C gene cluster on pig chromosome 10: possible associations with reproductive traits

**DOI:** 10.1186/1746-6148-2-28

**Published:** 2006-09-13

**Authors:** Dan J Nonneman, Tommy H Wise, J Joe Ford, Larry A Kuehn, Gary A Rohrer

**Affiliations:** 1 USDA-ARS, U.S. Meat Animal Research Center, Clay Center, Nebraska, 68933, USA

## Abstract

**Background:**

The rate of pubertal development and weaning to estrus interval are correlated and affect reproductive efficiency of swine. Quantitative trait loci (QTL) for age of puberty, nipple number and ovulation rate have been identified in Meishan crosses on pig chromosome 10q (SSC10) near the telomere, which is homologous to human chromosome 10p15 and contains an aldo-keto reductase (AKR) gene cluster with at least six family members. AKRs are tissue-specific hydroxysteroid dehydrogenases that interconvert weak steroid hormones to their more potent counterparts and regulate processes involved in development, homeostasis and reproduction. Because of their location in the swine genome and their implication in reproductive physiology, this gene cluster was characterized and evaluated for effects on reproductive traits in swine.

**Results:**

Screening the porcine CHORI-242 BAC library with a full-length *AKR1C4 *cDNA identified 7 positive clones and sample sequencing of 5 BAC clones revealed 5 distinct AKR1C genes (*AKR1CL2 *and *AKR1C1 *through *4*), which mapped to 126–128 cM on SSC10. Using the IMpRH_7000rad _and IMNpRH2_12000rad _radiation hybrid panels, these 5 genes mapped between microsatellite markers *SWR67 *and *SW2067*. Comparison of sequence data with the porcine BAC fingerprint map show that the cluster of genes resides in a 300 kb region. Twelve SNPs were genotyped in gilts observed for age at first estrus and ovulation rate from the F8 and F10 generations of one-quarter Meishan descendants of the USMARC resource population. Age at puberty, nipple number and ovulation rate data were analyzed for association with genotypes by MTDFREML using an animal model. One SNP, a phenylalanine to isoleucine substitution in *AKR1C2*, was associated with age of puberty (p = 0.07) and possibly ovulation rate (p = 0.102). Two SNP in *AKR1C4 *were significantly associated with nipple number (p ≤ 0.03) and another possibly associated with age at puberty (p = 0.09).

**Conclusion:**

AKR1C genotypes were associated with nipple number as well as possible effects on age at puberty and ovulation rate. The estimated effects of AKR1C genotypes on these traits suggest that the SNPs are in incomplete linkage disequilibrium with the causal mutations that affect reproductive traits in swine. Further investigations are necessary to identify these mutations and understand how these AKR1C genes affect these important reproductive traits.

The nucleotide sequence data reported have been submitted to GenBank and assigned accession numbers [GenBank:DQ474064–DQ474068, GenBank:DQ494488–DQ494490 and GenBank:DQ487182–DQ487184].

## Background

In swine, the rate of pubertal development and successful pregnancy in gilts affects the efficient management of breeding females. Selection for growth rate and leanness in modern commercial pigs has resulted in a delay in the onset of puberty [1]. Age at puberty and weaning to estrus interval (WEI) are positively correlated [2] and the primary reason for culling sows is failure to return to estrus after weaning. Quantitative trait loci (QTL) have been identified for age of puberty in the pig on different chromosomes [3,4]. One of the QTL regions is located on the long arm of pig chromosome 10 (SSC10q) near the telomere, which is homologous to human chromosome 10p15 [5,6]. The q-arm of pig chromosome 10 also has QTL for ovulation rate [4] and number of nipples [7-10]. In the human, an aldo-keto reductase (AKR) gene family (AKR1C) has been identified near the telomere on chromosome 10p15 that contains at least six aldo-keto reductase family 1, member C genes [11]. The homologous region on mouse chromosome 13 contains a cluster of eight or nine AKR1C genes [12]. These duplicated genes maintain a high degree of sequence similarity, but differ greatly in their substrate specificity and tissue expression patterns.

The AKR superfamily are monomeric oxidoreductases that catalyze the NADP(H)-dependent reduction of a wide variety of substrates, ranging from steroids, prostaglandins, bile acids, carbohydrates and xenobiotics [13]. AKRs are also thought to deactivate damaging reactive oxygen species like carbonyl compounds from lipids and proteins leading to their elimination [14,15]. Aldo-keto reductases interconvert weak androgens, estrogens, progestins, mineralocorticoids and glucocorticoids to their more potent counterparts by catalyzing the reduction and oxidation of keto- and hydroxysteroids, respectively, thereby regulating a wide range of physiological processes involved in development, homeostasis and reproduction [16]. In this manner, AKRs regulate the occupancy and transactivation of several steroid receptors in target tissues leading to transcription of hormone-responsive genes [13]. These steroid substrates can also act directly through "non-genomic" effects, such as, formation of neuroactive steroids and activation of ion channels, G-protein-coupled receptors and several kinase signalling pathways. The products of AKR activity have been implicated in prostate disease, breast cancer, obesity, polycystic ovary disease and delay in the onset of puberty in humans [17-22]. Because of their location in the swine genome and their implication in directing reproductive physiology, this gene cluster was characterized and evaluated for affecting age at puberty in the pig.

## Results

### Identification of AKR1C genes

A genomic amplicon spanning exons 4 and 5 of *AKR1C4 *(Genbank accession number AF473815; [5]) probed against one third of the porcine RPCI-44 BAC library identified 3 clones (62L11, 69L21 and 125A17). A full-length cDNA for *AKR1C4 *(TC200328, The Institute for Genomic Research (TIGR) [23] probed against one third of the porcine CHORI-242 BAC library identified seven clones (203C8, 204L24, 226I21, 264H20, 275P11, 315D4 and 319P22). Three of these clones (203C8, 226I21 and 264H20) are represented in contig 10007 of pig BAC fingerprint map [24]. Southern analysis of a *Bam*HI digest of 8 clones from both libraries using the same cDNA probe showed 5–9 shared bands and dissimilar banding patterns. Five of these BACs from the CHORI-242 library (203C8, 226I21, 264H20, 315D4 and 319P22) were digested with *Bam*HI and *Hind*III and subcloned for sample sequencing. One BAC clone (CHORI-242-203C8) that contained at least 4 AKR1C genes was nebulized and subcloned into pBluescript to obtain more coverage. The complete *AKR1C4 *gene was PCR cloned by amplification using exon primers and the RPCI44-125A17 BAC clone as template. Exon sequences for *AKR1CL2*, *AKR1C1, AKR1C2 *and *AKR1C3 *were found in 4 of the 5 CHORI-242 BAC subclone libraries (203C8, 226I21, 315D4 and 319P22) and *AKR1C4 *sequence was found in CHORI-242-264H20 subclones exclusively. *AKR1C2 *was found in both sets of BACs in the overlapping region. Sequences corresponding to *AKR1CL1 *were not found in BAC subclones. The promoter region of *AKR1C4 *was cloned by probing *Bam*HI/*Bgl*II digests of the RPCI BACs with a fragment containing exon 1 and part of intron 1. The promoter contained LSF (late SV40 factor), ERE (estrogen response element) and multiple SP1 and MYC sites [Genbank: DQ494489]. A 3900 bp contig from BAC clone CHORI-242-203C8 upstream of *AKR1CL2 *contained promoter elements CCAAT, SP1, ETS, GATA, NF-1 and ERE [Genbank: DQ494488] [25].

### Identification of cDNAs for AKR1C

**Figure 1:**
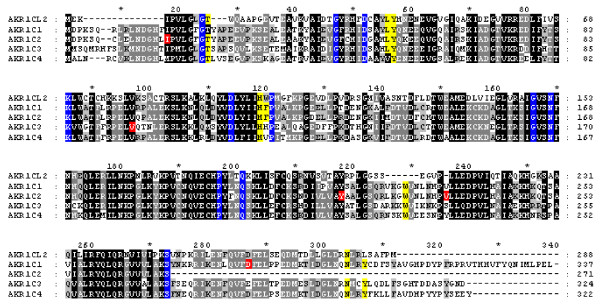
**Alignment of pig AKR1C mRNAs**. Conserved residues T24, L54, Y55, H117, F118, W227, N306 and Y310 involved in substrate binding are highlighted in yellow [52] and residues highlighted in blue are invariant residues found in all AKRs [53]. Residues Asp274Asn in *AKR1C1*, Ile16Phe, Tyr216Asn, Val234Phe in *AKR1C2 *and Val97Phe in *AKR1C3 *in red are non-synonymous amino acid polymorphisms identified in cDNA and genomic sequence of Meishan and White composite pigs. Dashes indicate residues that are absent, shading indicates identity and asterisks represent 10 bp increments.

**Figure 2:**
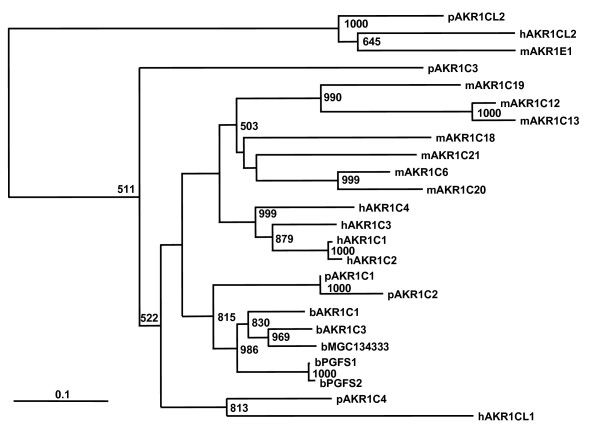
**Phylogenetic relationship of AKR1C family members of human, mouse, cow and pig**. Multiple sequence alignment was performed by ClustalW and neighbor-joining tree of full-length amino acid sequences is shown. Species is designated by a lower case letter preceding the AKR1C gene family member (h = human; b = bovine; p = porcine and m = mouse). Numbers at branches represent 1000 bootstrapped datasets and scale bar represents substitutions per site. Human genes are from the AKR1C cluster on chromosome 10p15, mouse and bovine genes are from chromosomes 13 in both species and the five pig genes on SSC10q described in this study. In general, except for *AKR1CL2*, paralogues within species are more related than are homologues among species.

TIGR contigs were identified for four of the five AKR1C genes identified from BAC subclone sequence; only *AKR1C2 *was not represented in EST libraries sequenced or in contigs assembled by TIGR. These mRNAs were confirmed by RT-PCR of overlapping fragments or by completely sequencing individual clones. Amino acid and nucleotide homologies ranged from about 50–90% and 73–93%, respectively, among the pig AKR1C transcripts and the presence of active site residues common to aldo-keto reductases was conserved (Figure 1). No additional ESTs that would represent *AKR1CL1 *or other AKR1C genes were identified by sequence similarity analyses. Because of the high homology of these genes to all of the human AKR1C genes they were named by their relative position and conservation of amino acid sequence with human genes. The identity of pig *AKR1CL2 *could be confidently assigned; pig *AKR1CL2 *was most similar to human *AKR1CL2 *and mouse *Akr1e1 *(Figure 2) and had little similarity to other human *AKR1C *genes. The pig *AKR1C1 *followed by *AKR1C2 *was the most similar to human *AKR1C *genes (83–85%) and to bovine *AKR1C *genes; pig *AKR1C4 *was more closely related to human *AKR1C4 *and *AKR1CL1*. Except for *AKR1CL2*, paralogues were more similar within species than were homologues among species.

### Mapping and gene organization

**Figure 3:**
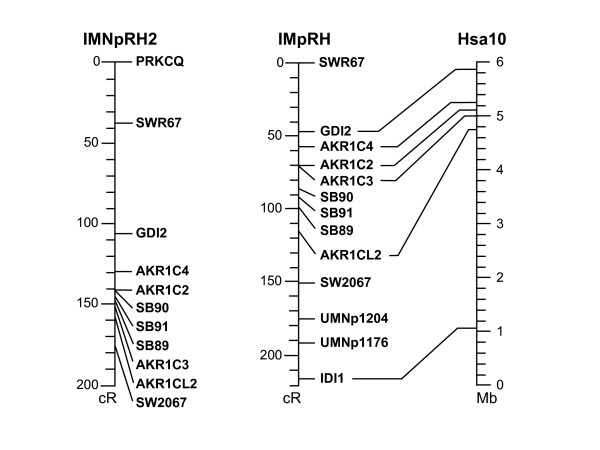
**Radiation hybrid maps of AKR1C genes on IMpRH_7000rad _and IMNpRH2_12000rad _panels**. The IMpRH map is aligned to human chromosome 10p15 (inverted).

**Figure 4:**
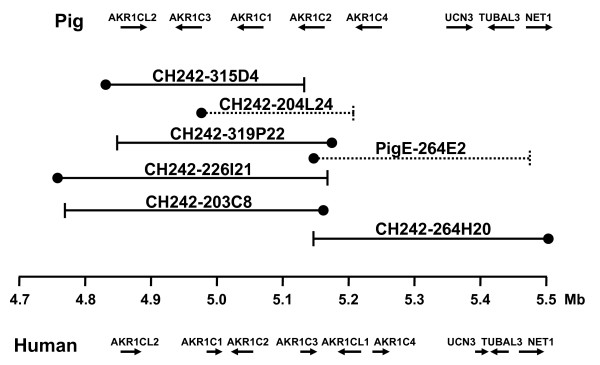
**BAC contig map of region containing pig AKR1C genes**. Location and orientation of pig genes is shown above. BAC clone ends are represented as ● for SP6 primer sequence and ⊢ as the T7 primer sequence end. Clones in dashed lined were not subcloned and sequenced but aligned by BAC-end sequences (BES). The human position on HSA10 is shown in megabases and gene order is shown below. Not all porcine BACs in this region are shown.

SNPs identified in three of the five genes (*AKR1CL2*, *AKR1C2 *and *AKR1C4*, [Genbank: BV102614, BV680543, AF473815, respectively]) and microsatellites SB88-91 [GenBank: DQ487182-84] found in BAC subclone sequences were linkage mapped to SSC10, positions 126–128 cM. The resolution of the map was not great enough to determine order of genes or markers. Four AKR1C genes (*AKR1CL2*, *AKR1C2*, *AKR1C3 *and *AKR1C4*) and microsatellite markers *SB89*, *SB90 *and *SB91 *(Table 1) were mapped using the IMpRH_7000rad _and IMNpRH2_12000rad _radiation hybrid panels and anchored with flanking genes (*PRKCQ, GDI2 *and *IDI1*) and microsatellite markers *SWR67 *and *SW2067 *(Figure 3). The resolution of the IMNpRH2_12000rad _panel was nearly the same as that of the IMpRH_7000rad _panel. Comparison of sample sequence data with mRNA sequences and the porcine BAC fingerprint map showed that the complete cluster of genes resides in two overlapping BAC clones that were subcloned, CHORI-242-203C8 and 264H20; clones 226I21, 315D4 and 319P22 completely overlap 203C8 (Figure 4). *AKR1CL2*, *AKR1C1*, *AKR1C2 *and *AKR1C3 *were all contained within CHORI-242 203C8 and microsatellite markers *SB89, SB90 *and *SB91 *were identified from this clone, as well. The gene order was determined to be *AKR1C4*, *AKR1C2*, *AKR1C1*, *AKR1C3 *and *AKR1CL2 *from centromere to telomere. BAC clone CHORI-242-264H20 contained *AKR1C2*, *AKR1C4*, the urocortin 3 (*UCN3*), tubulin alpha-like 3 (*TUBAL3*) and neuroepithelial cell transforming gene 1 (*NET1*) genes (Figure 4). The orientation of the AKR1C genes was determined by aligning cDNAs or BAC subclone contigs with BES on the BAC fingerprint map [24]. This region corresponds to about 630 kb of human sequence (4.8–5.5 Mb on HSA10) but is contained in only two porcine BAC clones, average insert size of 173 kb, suggesting that this region is about half the size in the pig. A portion of this reduction is possibly due to the lack of a pig homologue to human *AKR1CL1*, although it is possible that AKR1CL1 was missed in the BAC sequence survey.

### Expression of pig AKR1C genes in different adult tissues

**Figure 5:**
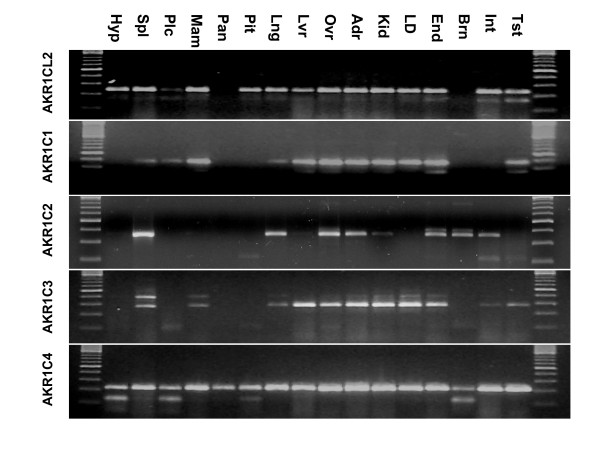
**RT-PCR amplification of pig AKR1C genes in different tissues from a purebred Meishan sow at day 25 of gestation and mature testis using gene-specific primers (Hyp, hypothalamus; Spl, spleen; Plc, placenta; Mam, mammary tissue; Pan, pancreas; Pit, pituitary; Lng, lung; Lvr, liver; Ovr, ovary; Adr, adrenal; Kid, kidney; LD, longissimus dorsi muscle; End, endometrium; Brn, brain; Int, small intestine; Tst, testis)**. Amplicon sizes are given in Table 1; marker is a 100 bp ladder.

Gene-specific cDNA for the five different AKR1C genes was amplified from 16 tissues (Table 1). Six tissues expressed all five genes tested (spleen, lung, ovary, adrenal, kidney, and endometrium) and *AKR1CL2 *and *AKR1C4 *were the most widely expressed genes (Figure 5). *AKR1CL2 *was expressed in all tissues except pancreas and brain. Unlike human *AKR1C4*, pig *AKR1C4 *expression was not specific to the liver but was expressed in all tissues and *AKR1C2 *was the only other AKR1C gene expressed in brain. These expression patterns were also reflected by the number of clones and tissue source of the EST libraries from which these cDNAs were identified [23].

### Association of SNPs with phenotypic traits

Polymorphisms were identified from RT-PCR sequences of Meishan and White composite endometrium cDNA. SNPs identified in the coding region were genotyped across generations F8 and F10 of the resource population. Additional SNPs were found by sequencing genomic DNA from animals of similar breed types and were chosen based on their frequency and potential for being a non-synonymous mutation (Table 2 and Additional File 1). Twelve SNPs were genotyped for association with reproductive traits. Three of these were in the *AKR1C2 *coding region, one was in *AKR1CL2*, and eight were the *AKR1C4 *gene, including one in the promoter region (Table 2). One SNP (49422_42), an isoleucine to phenylalanine substitution in *AKR1C2*, was associated with age of puberty (p = 0.07) and possibly ovulation rate (p = 0.102). Another SNP in *AKR1C4 *(49431_198) was possibly associated with age at puberty (p = 0.093; Table 3). Two other SNP were significantly associated with nipple number (p ≤ 0.03; Table 3).

## Discussion

As in other species, this gene cluster is conserved in the pig, although individual family members have undergone some sequence divergence and specialization of tissue expression, possibly due to duplication of function. Because of high sequence similarity of the genes within species and divergence among species, it is difficult to definitively assign homologues for all members of the gene family and not all genes are represented in the pig (i.e., *hAKR1CL1*) that are found in human or mouse. Gene duplication usually results in tandem duplication of genes or segments along the chromosome [26] and gene conversion can result in a species paralogues being more closely related than homologues among species [27]. Because these genes are expressed in a multitude of tissues and the expression patterns in pig tissues do not differentiate these genes with expression patterns described in human or mouse, assignment of homologues is even more complicated. In addition, the orientation of genes in this cluster is not identical to human or mouse gene order, while gene order of flanking genes (UCN3, TUBAL3, and NET1) is conserved [11]. As more species are fully sequenced, a clearer picture of the evolutionary process of this gene family can be drawn.

Because this pluripotent family of enzymes regulates steroid hormone action in a tissue-specific manner, they are compelling positional candidates for regulating reproductive functions [16,28]. Steroid metabolites of AKR1C enzymes rise at the onset of puberty [22,29] presumably due to increased substrate and enzyme activity. The onset of puberty is marked by hormonal changes directed by neuronal signals that result in activation of the hypothalamic-pituitary-gonadal axis and reproductive maturity [30]. Central to behavioral and gonadal maturity is the release of gonadotropin releasing hormone (GnRH) followed by synthesis and secretion of luteinizing hormone (LH) and follicle stimulating hormone (FSH). An LH surge in turn is essential for stimulating the cascade of events leading to ovulation [31]. The gonadal steroid 3alpha-hydroxy-4-pregnen-20-one (3 alpha HP) produced from progesterone by AKR1C inhibits GnRH activity on gonadotropes and suppresses FSH release from pituitary cells [32]. Modulation of the GnRH pulse frequency could therefore cause variation in the timing of puberty. Furthermore, as GnRH regulates FSH and LH release, it is possible that AKR1C activity may affect the number of ova shed during an estrus. FSH secretion stimulates the development of antral follicles and FSH levels are greater in some lines of gilts with higher ovulation rate [33,34].

QTL for nipple number have been identified on SSC10q and two SNPs in *AKR1C4 *were significantly associated with nipple number. Some of these QTL are more proximally located on SSC10q [8-10], but one identified in a Meishan/Pietrain cross maps to the same location as the AKR1C gene cluster [7]. Because androgen and antiandrogen treatment in rodents alters nipple development and retention [35], and treatment of rats with an inhibitor of 5α-reductase during gestation inhibits male nipple regression [36], a role for dihydrotestosterone (DHT) is implicated in normal nipple development. AKRs convert DHT, a preferred substrate, to the less active androgen 3α-androstanediol, thereby regulating steroid responsiveness in target tissues [13] such that variation in AKR1C activity could affect nipple development in the pig. *AKR1C4 *is the most catalytically active isoform for DHT reduction in human [28] and has high expression in mammary tissue (Figure 5). Because it is ubiquitously expressed and ESTs have been identified in porcine embryonic libraries [23], it is probably expressed during embryonic development of mammary tissue.

Considerable support for an association of the AKR1C genotypes with nipple number was detected as well as some indication of an effect on age at puberty and possibly ovulation rate. Age of puberty and ovulation rate at a specific age are negatively correlated traits in Meishan pigs because ovulation rate increases from puberty to later estrus cycles [37] and animals that reach puberty earlier will have had more cycles at the time of measurement and greater number of ova shed; however, this increase in ovulation rate is less dramatic in occidental pigs. Because there was no selection performed on these animals and this is an area of increased recombination, it is assumed that recombination has greatly reduced linkage disequilibrium in this region, facilitating fine-mapping of reproductive traits in advanced generations (F8 and F10) of this population. While genetic variation in this region appears to affect reproductive traits in swine there is little evidence that the SNP markers tested are causative. Rather, the estimated effects of AKR1C genotypes show an overdominant effect on reproductive traits suggesting that these SNPs may be in incomplete linkage disequilibrium with the causal mutations or possibly there are multiple causative SNPs within this region acting in repulsion. Further investigations are necessary to identify the causal mutations and understand the role AKR1C genes have on these important reproductive traits.

## Conclusion

Variation in the aldo-keto reductase gene cluster on pig chromosome 10 may be associated with age of puberty, nipple number and ovulation rate in swine. Future studies will determine if this variation will be useful for selection of breeding females with greater reproductive efficiency in industry populations.

## Methods

### cDNA synthesis, amplification and sequencing

Porcine cDNAs for aldo-keto reductases (AKRs) were identified from EST sequences deposited in GenBank and assembled at The Institute for Genomic Research (TIGR) [23] or by homology of porcine genomic BAC sequence to human AKRs. Tissues from adult purebred and composite breed animals were collected in RNAlater (Ambion, Austin, TX) and homogenized in Trizol (Invitrogen, Carlsbad, CA) as the source for total RNA synthesis of RT-PCR template for identification of sequence variation. Tissues from a purebred Meishan sow collected at day 25 of gestation and testis from a mature boar were used for differential expression of AKR1C transcripts. cDNA was synthesized with M-MLV reverse transcriptase (Promega, Madison, WI) using 2 μg of total RNA from endometrium, placenta, testis, ovary, liver, lung, adrenal, kidney, spleen, pituitary, hypothalamus, brain, pancreas, small intestine, skeletal muscle and mammary tissue. These reactions were run for 35 cycles with 20 ng of template as described below. Sequences were extended using 3'-RACE or RT-PCR from exon sequences identified in BAC subclones. Full-length cDNA clones were obtained by iterative screening and self-ligation of inverse PCR (SLIP) [38] of the MARC 1PIG and 2PIG primary libraries before normalization [39].

### BAC screens and subclone libraries

Filters from the RPCI-44 and CHORI-242 porcine BAC libraries were screened using a random-primed nearly full-length cDNA of porcine *AKR1C4 *as probe. The probe was prepared by PCR of the MARC 2PIG library using primers in exons 1 and 9 and radioactive random-primed labeled (Megaprime DNA Labeling System, Amersham, Piscataway, NJ). Positive clones were grown in 100 ml cultures and processed for BAC DNA using a midi-prep column (Marligen Biosciences, Ijamsville, MD). The BACs were digested with *Bam*HI and *Hind*III separately and subcloned into pBluescript. One 96-well plate of clones was processed for each BAC and enzyme combination and sequenced with T3 and T7 primer. One CHORI-242 BAC (203C8) was sheared with a nebulizer (Invitrogen, Carlsbad, CA), end-repaired, then cloned into pBluescript and four 384-well plates of clones were sequenced.

### PCR and sequencing

Primer pairs for amplification of genomic DNA were designed from porcine AKR1C cDNA sequences and genomic sequence obtained during this study using Primer 3; code available at the Primer 3 Software website [40]. PCR was performed in a PTC-225 DNA engine (MJ Research Inc, Watertown, Mass) using 0.25 U Hot Star^® ^*Taq *polymerase (Qiagen, Valencia, CA, USA), 1× of supplied buffer, 1.5 mM MgCl_2_, 200 μM dNTPs, 0.8 μM each primer, and 100 ng of genomic DNA in 25 μl reactions. Five μl of the PCR reaction was electrophoresed in 1.5% agarose gels to determine quality of amplification and the remainder was prepared for sequencing. Chromatograms were imported into the MARC database, bases called with Phred, assembled into contigs with Phrap, polymorphisms identified using Polyphred, and assessed using Consed [41].

### SNP genotyping

SNPs were mapped using a primer extension assay on the Sequenom MassArray™ system (San Diego, CA, USA). Ten μl PCR reactions contained 10 ng of genomic DNA, 0.25 U HotStar *Taq*, 1× of supplied buffer, 1.5 mM MgCl_2_, 200 μM dNTPs, and 0.4 μM forward and reverse tailed primers. The primer extension reaction used 0.6 μM of probe primer and was performed according to the manufacturer's recommendations for hME chemistry (Sequenom, San Diego, CA, USA).

### Radiation hybrid and linkage mapping

Genes were mapped using the 118-clone INRA-University of Minnesota porcine Radiation Hybrid IMpRH_7000rad _panel and the 90-clone IMNpRH2_12000rad _panel [42,43]. Primers used were described above, or designed from sequences obtained from subclone sequences. Amplifications were performed in 15 μl PCR in duplicate using 12.5 ng panel DNA, 1.5 mM MgCl_2_, 200 μM dNTPs, 1 μM each primer, 0.25 U Hot Star^® ^*Taq *and 1× of supplied buffer. The PCR mixture was held at 94°C for 15 min, and cycled 40 times at 94°C for 20 sec, held at the indicated annealing temperature for 30 sec and extension at 72°C for 45–60 sec, followed by a final extension at 72°C for 5 min. One half of the reaction was loaded on 2% agarose gels and manually genotyped. Data were analyzed for two-point and multipoint linkage with the IMpRH mapping tool [44] and submitted to the IMpRH database [45]. Carthagene [46,47] was used to estimate multipoint marker distance and order using all public markers on chromosome 10 in the IMpRH database [45] for the IMpRH_7000rad _panel and those developed in this study for the IMNpRH2_12000rad _panel to approximate position of mapped markers. Markers run on the IMpRH_7000rad _panel have been submitted to the IMpRH public database.

Linkage analyses were performed as described [48] where TWOPOINT analyses were used to indicate the chromosome linkage group and the ALL, FLIPS and FIXED options were used to determine the multipoint position of the marker (CRIMAP v2.4). Multipoint locations for all mapped markers are based on the latest published swine genetic map [49].

### Animals and resource population

Genomic DNA from parents of the MARC reference family (a White composite boar and seven crossbred sows) was used to identify SNPs. Phenotypes and genotypes were collected from animals of generations 8 and 10 (F8 and F10) produced from the original resource population [4] used to identify QTL. The original animals were from reciprocal backcrosses of 10 purebred Meishan and 10 White composite (composed of Chester White, Landrace, Large White and Yorkshire). The half-Meishan F4 animals were crossed with a Landrace-Large White composite reducing the Meishan influence to one quarter. This population has been *inter se *mated since the F5 generation and maintained at 30 litters/year. Procedures for the handling of animals complied with those specified in the *Guide for the Care and Use of Agricultural Animals in Agricultural Research and Teaching *(1999) [1st rev. ed. Savoy, IL: Federation of Animal Science Societies; 1999].

### Phenotypic data and statistical methods

The F8 gilts were observed for first estrus beginning when the oldest gilts reached 120 days of age as described by Rohrer et al. [4], and ovulation rates were determined by counting corpora lutea on the ovaries at slaughter after the third estrus in gilts from the F8 and F10 generations. Genotype probabilities were calculated for all animals using an extended version of GenoProb [50]. Association analyses between phenotypes and genotypes were conducted using MTDFREML [51]. The model fitted included fixed effects for contemporary group and regressions on SNP marker genotype probabilities (i.e., probability of an animal being aa, aA, or AA). Random effects included the animal's polygenic breeding value and residual error. Each marker was analyzed separately. No adjustments to reported p-values were made for multiple comparisons. Twelve SNP markers were analyzed for three traits; age of puberty (191 observations), ovulation rate (233 observations) and nipple number (1144 observations).

### Phylogenetic analysis

AKR1C peptide sequences were aligned with ClustalX, the distance matrix constructed with the program PRODIST using a Dayhoff PAM matrix model, and a neighbor-joining tree constructed in PHYLIP (v 3.65). The tree was constructed with the program NEIGHBOR and rooted at a mid-point with the program RETREE. Bootstrap values were derived from 1000 pseudo-datasets generated in SEQBOOT. The tree was viewed in TREEVIEW (v 1.6.6).

## Competing interests

The author(s) declare they have no competing interests.

## Authors' contributions

DN oversaw the laboratory work and drafted the manuscript. TW and JJF collected phenotypic data, GAR participated in design of the study, interpretation of results and collection of genotypic data and map information, and LAK performed detailed statistical analysis. All authors contributed to final preparation of the manuscript.

## Supplementary Material

Additional File 1 SNP Summary. The data provided represent the sequences, associated primers, GenBank accession numbers and SNPs identified in pig aldo-keto reductase 1C genes. Click here for file

## Figures and Tables

**Table 1:** Primers used for mapping and tissue-specific expression of pig AKR1C genes.

Marker	GenBank Accession^1^	TIGR Contig	Forward primer (5'→3')	Reverse primer (5'→3')	Application	Size (bp)
AKR1CL2	DQ494488	TC204964	gcgtctattacctccttgcatc	acctcgttctcattgtggtaca	RH mapping	441
AKR1C1	BV677932	TC223177	ggagtgttttctctccatggtc	cctatttcaatgatccacacga	RH mapping	322
AKR1C2	BV680531	None	atcccaaagccagtgtctg	ttacatctgatctgccaaggtg	RH mapping	348
AKR1C3	DQ494490	TC200330	taagggcagacacagcttgata	tggaatacaaaactgaggcaga	RH mapping	279
AKR1C4	BV680541	TC200328	atcatcacctagggtcaacaattc	gagctcccgctcagtactcttcag	RH mapping	345
SB89 microsatellite	DQ487182	None	ttccacaccctctccttctcta	tgaaattcagttggaaagttttga	RH/linkage map	150
SB90 microsatellite	DQ487183	None	gggaattccatatgccatgagt	aagctgcagaaagagggaagta	RH/linkage map	117
SB91 microsatellite	DQ487184	None	aaattgaggaggagggaacagt	gtcaacaagatatggcctgta	RH/linkage map	165
AKR1CL2	DQ474064	TC204964	acccataccttacgcagaagaa	aaattctcaagaatccgctttg	RT-PCR	240
AKR1C1	DQ474065	TC223177	cgatggtcacttcattcctgta	agtgcttttccgttttcatctg	RT-PCR	380
AKR1C2	DQ474066	None	tccttcgaccagagttggtcca	catttgacttgcagaaatccag	RT-PCR	364
AKR1C3	DQ474067	TC200330	ccattcagatgaaaattgcaga	ctcgttcttgcacttctccat	RT-PCR	280
AKR1C4	DQ474068	TC200328	aagacagagtgagcccagccg	cacctcctcctcgttttcatag	RT-PCR	215

^1^Accession number is for genomic DNA for RH/linkage markers and mRNA sequence for RT-PCR.

**Table 2:** SNPs identified in AKR1C genes genotyped in 1/4 Meishan F8 and F10 population.

					Allele frequencies^3^			
						
Assay	Gene	Accession^1^	Location^2^	Polymorphism	F8 & F10	MS	WC	Polymorphic site^4^
33391_261 C/T	AKR1CL2	BV102614	Intron 1		T = 0.63	T = 0.21	T = 0.81	ccctgcacag**Y**agcgcgggca
49422_42 A/T	AKR1C2	BV680531	nt 179	Ile16Phe	A = 0.77	A = 0.50	A = 1.00	cggtcacctc**W**ttcctgtact
36962_196 A/T	AKR1C2	DQ474066	nt 779	Tyr216Asn	A = 0.62	A = 0.17	A = 0.89	tttggttgcc**W**atgctgccct
36962_681 A/G	AKR1C2	DQ474066	nt 833	Val234 Ile	G = 0.85	ND	ND	aaaccaccca**R**ttctcttgg
31063_1495 G/T	AKR1C4	BV680526	promoter	ERE site	T = 0.55	T = 0.10	T = 0.94	tcagagtact**K**accttgacca
31503_2415 A/G	AKR1C4	BV680525	Exon 1	silent	G = 0.81	G = 1.00	G = 0.67	tgagtagtgg**R**gaagagacgt
27648_208 C/T	AKR1C4	BV680527	Exon 1	silent	T = 0.67	T = 0.36	T = 0.86	agctgaatga**Y**gggcacctca
20502_681 C/T	AKR1C4	BV680537	Intron 2		C = 0.57	C = 0.17	C = 0.92	cttttagcta**Y**tgatgtttmc
20502_722 C/T	AKR1C4	BV680537	Intron 2		C = 0.78	C = 0.50	C = 1.00	aaatcayaag**Y**catttttaat
14984_99 G/T	AKR1C4	BV680523	Intron 4		T = 0.56	ND	ND	aaatgtgggt**K**tgatccctgg
36969_772 C/T	AKR1C4	BV680543	Exon 7	silent	C = 0.56	C = 0.25	C = 0.94	agaaacacaa**Y**agaagcccag
49431_198 C/T	AKR1C4	BV680533	Intron 8	Splice site	T = 0.58	T = 0.50	T = 0.81	ttcccttcct**Y**ycaggtttttr

^1^Accession numbers beginning with BV are sequences submitted to dbSTS.

^2^Location refers to nucleotide position in cDNA.

^3^Allele frequencies were calculated from F8 and F10 animals genotyped (ranging from 238–420 animals), and founder Meishan (MS) and founder White composite (WC) animals (7–10 animals each) in the original resource population; ND = not determined.

^4^Polymorphisms are identified by IUB code with flanking genomic sequence.

**Table 3:** Tests of significance and estimated effects of AKR1C genotypes on reproductive traits in F8 and F10 animals.

Assay	Gene	Age at puberty^1^	Ovulation rate^2^	Number of nipples^3^
33391_261	AKR1CL2	p > 0.15^4^	p > 0.15	0.148 (TT = 0; CT = 0.170; CC = -0.077)
49422_42	AKR1C2	0.070 (TT = 0; AT = 13.46; AA = 6.21)	0.102 (TT = 0; AT = 1.54; AA = 1.15)	p > 0.15
31063_1495	AKR1C4	p > 0.15	p > 0.15	0.064 (TT = 0; GT = 0.280; GG = 0.206)
27648_208	AKR1C4	p > 0.15	p > 0.15	0.024 (CC = 0; CT = 0.476; TT = 0.276)
20502_681	AKR1C4	p > 0.15	p > 0.15	0.030 (CC = 0; CT = 0.304; TT = 0.119)
14984_99	AKR1C4	p > 0.15^4^	p > 0.15	0.058 (TT = 0; GT = 0.284; GG = 0.180)
36969_772	AKR1C4	p > 0.15	p > 0.15	0.106 (CC = 0; CT = 0.263; TT = 0.199)
49431_198	AKR1C4	0.093 (CC = 0; CT = 6.53; TT = -2.17)	p > 0.15	p > 0.15

^1^The analysis for age at puberty contained 191 observations, mean = 195 ± 27 days (mean ± std dev), range = 135 – 266.

^2^233 observations for ovulation rate, mean = 13.7 ± 2.4 corpora lutea, range = 9 – 21.

^3^1144 observations for nipple number, mean = 14.6 ± 1.06 nipples, range = 11 – 19. Genotype probabilities were calculated for all F8 and F10 animals in the population using GenoProb [50].

^4^Values indicate p-value and estimated genotype effects in parentheses. The allele frequencies for F8 and F10 animals are given in Table 2.
